# Genetic Variation in an Individual Human Exome

**DOI:** 10.1371/journal.pgen.1000160

**Published:** 2008-08-15

**Authors:** Pauline C. Ng, Samuel Levy, Jiaqi Huang, Timothy B. Stockwell, Brian P. Walenz, Kelvin Li, Nelson Axelrod, Dana A. Busam, Robert L. Strausberg, J. Craig Venter

**Affiliations:** J. Craig Venter Institute, Rockville, Maryland, United States of America; University of California, San Diego and The Scripps Research Institute, United States of America

## Abstract

There is much interest in characterizing the variation in a human individual, because this may elucidate what contributes significantly to a person's phenotype, thereby enabling personalized genomics. We focus here on the variants in a person's ‘exome,’ which is the set of exons in a genome, because the exome is believed to harbor much of the functional variation. We provide an analysis of the ∼12,500 variants that affect the protein coding portion of an individual's genome. We identified ∼10,400 nonsynonymous single nucleotide polymorphisms (nsSNPs) in this individual, of which ∼15–20% are rare in the human population. We predict ∼1,500 nsSNPs affect protein function and these tend be heterozygous, rare, or novel. Of the ∼700 coding indels, approximately half tend to have lengths that are a multiple of three, which causes insertions/deletions of amino acids in the corresponding protein, rather than introducing frameshifts. Coding indels also occur frequently at the termini of genes, so even if an indel causes a frameshift, an alternative start or stop site in the gene can still be used to make a functional protein. In summary, we reduced the set of ∼12,500 nonsilent coding variants by ∼8-fold to a set of variants that are most likely to have major effects on their proteins' functions. This is our first glimpse of an individual's exome and a snapshot of the current state of personalized genomics. The majority of coding variants in this individual are common and appear to be functionally neutral. Our results also indicate that some variants can be used to improve the current NCBI human reference genome. As more genomes are sequenced, many rare variants and non-SNP variants will be discovered. We present an approach to analyze the coding variation in humans by proposing multiple bioinformatic methods to hone in on possible functional variation.

## Author Summary

Characterizing the functional variation in an individual is an important step towards the era of personalized medicine. Protein-coding exons are thought to be especially enriched in functional variation. In 2007, we published the genome sequence of J. Craig Venter. Here we analyze the genetic variation of J. Craig Venter's exome, focusing on variation in the coding portion of genes, which is thought to contribute significantly to a person's physical make-up. We survey ∼12,500 nonsilent coding variants and, by applying multiple bioinformatic approaches, we reduce the number of potential phenotypic variants by ∼8-fold. Our analysis provides a snapshot of the current state of personalized genomics. We find that <1% of variants are linked to any known phenotypes; this demonstrates the dearth of scientific knowledge for phenotype-genotype associations. However, ∼80% of an individual's nonsynonymous variants are commonly found in the human population and, because phenotypic associations to common variants will be elucidated via genome-wide association studies over the next few years, the capability to interpret personalized genomes will expand and evolve. As sequencing of individual genomes becomes more prevalent, the bioinformatic approaches we present in this study can be used as a paradigm to pursue the study of protein-coding variants for the genomes of many individuals.

## Introduction

Genetic variation in the protein-coding portion of genes is of significant interest in the study of human health. The focus on coding exons, or the ‘exome’, is due to the common belief that the exome harbors the most functional variation [1]. This is based on the observation that mutations that cause Mendelian diseases occur primarily in genes [1],[2]. Mutations that cause amino acid substitutions, including changes to nonsense codons, in their respective genes are the most frequent type of disease mutation (∼60%) [1]. In addition, small indels in genes account for almost a quarter of the mutations in Mendelian disease [1],[2]. Meanwhile, less than 1% of Mendelian disease mutations have been found in regulatory regions. For complex diseases, such as Alzheimer's, obesity, and heart disease, it is unknown how much variation in genes will contribute to disease, compared to variation in regulatory regions [3],[4].

There have been many efforts to re-sequence genes to identify and characterize gene variation in humans [5]–[11]. One of the proposals of the 1000 Genomes Project, an international collaboration that aims to sequence one thousand genomes, focuses specifically on re-sequencing coding exons [12]. Additionally, many groups are developing technology for high-throughput resequencing of exons [13]–[15].

Because there has been much progress in sequencing individual human genomes [16]–[20], our understanding of functional variation is an important step towards an era of personalized medicine, where a doctor could inform patients' of their disease susceptibilities based on their genome sequences. If the exome harbors much of the functional variation responsible for a person's phenotype, then identification and characterization of the individual's variation in the exome could enable individualized genomics.

In this study, we focus our analysis on the exome of an individual human by providing a detailed characterization of the variants in protein-coding regions. We present the analysis of the coding variants in an exome from a diploid human genome assembly, which was termed HuRef [16]. This paper analyzes the different types of nonsilent coding variants. There are ∼12,500 coding variants that change protein sequence in the HuRef genome. We show that most of the variation in this individual is common and appears to be functionally neutral. Furthermore, we are able to reduce the ∼12,500 coding variants down to ∼1,600 variants that potentially affect protein function and may be involved in phenotypic effects. To the best of our knowledge, this is the first analysis of the exome of an individual human, and may serve as a benchmark for future studies on the variation in human exomes.

There are several aspects to this study. The first is to describe a snapshot of what personalized genomics means today. If a person was to have his genome sequenced today, we show what insights about the individual could be gleaned from the protein coding component alone. Another aspect is that we can use the HuRef variants to improve the current NCBI reference genome, which will simplify future analysis of additional genomes. The final aspect is how one could characterize the variation obtained from sequencing many individual human genomes, and the approaches that could be developed to mitigate these studies. This is our first glimpse of an individual's human genome and as additional genomes are sequenced, many rare variants and non-SNP variants will be discovered. From this first exome, we can see what challenges one might encounter and propose approaches to face these challenges. Thus we present one approach for the analysis of coding variation in a human by detecting different trends for each variant type and demonstrating what phenotypes can be interpreted with our current knowledge.

## Results

### Nonsynonymous SNPs

This individual has 10,389 nsSNPs, of which 5,604 are heterozygous and 4,785 are homozygous (Table 1), where homozygous variants are loci where the alleles differ from the NCBI reference genome, but are the same within the HuRef assembly. It has been previously estimated that the number of heterozygous nsSNPs in an individual ranges from 24,000 to 40,000 [6]; our observed value of 5,604 is much less than this. This estimate was based on the nonsynonymous substitution rate based on a small number of genes, and extrapolating this value across all genes. The overestimate is partly due to the assumption of the human genome having 45,000–100,000 genes, but even if we assume the human genome has 20,000–30,000 genes, the estimate remains 1.5–2× higher than what we report. Possible explanations for the discrepancy is that the substitution rate in genes is extremely variable due to differences in local rates of mutation and recombination [6]. Thus we believe our number to be more accurate because it examines all genes rather than extrapolating from a small gene set.

**Table 1:** Number of coding SNPs in HuRef.

Synonymous		10,413
Heterozygous	Novel	551
	dbSNP	5,183
Homozygous	Novel	98
	dbSNP	4,581
Nonsynonymous		10,389
Heterozygous*	Novel	557
	dbSNP	5,047
Homozygous	Novel	215
	dbSNP	4,570

*: All heterozygous novel nonsynonymous SNPs were manually inspected (see Methods).

The nsSNPs account for a little more than half of the coding SNPs in the diploid genome (Table 1). The 1∶1 ratio of nonsynonymous SNPs to synonymous SNPs agrees with previously published reports [6],[8]. Approximately 7% of the nsSNPs were not found in dbSNP and are thus novel. We expect novel SNPs to be rare [21]–[23] and hence observed on a single chromosome in an individual. This was affirmed with the observation that 72% of the novel nsSNPs are heterozygous.

### Most nsSNPs in an Individual Are Common

We wanted to find nsSNPs in this individual that may be undergoing negative selection. We use allele frequency in the human population as an indicator that a variant might be under negative selection. According to the theory of natural selection, functionally neutral mutations can reach high minor allele frequencies, whereas deleterious mutations will be selected against and remain rare in a population. Rare variants do not necessarily have to be deleterious; they can be recent mutational events. Variants that are neutral, slightly deleterious, or under positive selection can become common in a population.

**Figure 1:**
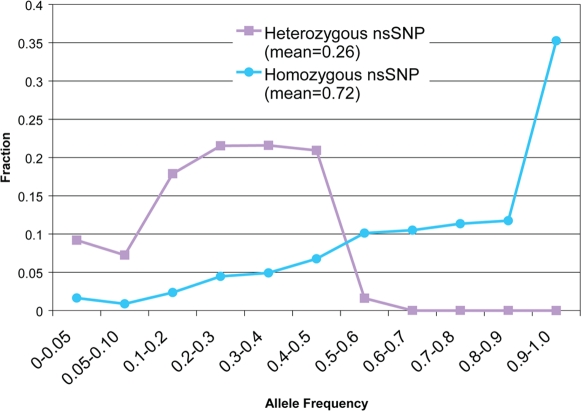
The allele frequencies of heterozygous and homozygous nsSNPs in HuRef. For heterozygous SNPs, the minor allele frequency is plotted. For homozygous nsSNPs, the frequency for the observed allele in HuRef is plotted.

To see what proportion of nsSNPs may be undergoing negative selection, we retrieved the allele frequencies of these nsSNPs from the HapMap Project [24],[25] (Figure 1). The majority of HuRef nsSNPs with known allele frequencies are common (> = 0.05). For 79% of the homozygous nsSNPs, the NCBI human genome has the minor allele (MAF<0.5). Therefore, the homozygous nsSNPs in HuRef tend to represent the major alleles in the human population and it is likely that the majority of these homozygous nsSNPs are neutral because they have reached such high frequencies. Also, 19% of the homozygous alleles in HuRef have an allele frequency of 1, which suggests that NCBI contains a rare or erroneous allele at these positions. The majority of HuRef heterozygous SNPs are also common with only 9% of the nsSNPs with known allele frequencies being rare (MAF<0.05).

Since we do not have allele frequencies for all the HuRef nsSNPs, we must estimate the proportion of rare nsSNPs (MAF<0.05) in this individual. A previous simulation has estimated that ∼28% of heterozygous SNPs in an individual are rare, but that study made assumptions about human population size and its growth [26]. For 67% of the nsSNPs with known HapMap allele frequencies, we know the exact number of rare nsSNPs (56 homozygous and 326 heterozygous). For the remaining 33% of nsSNPs with unknown allele frequencies, we can estimate the proportion of rare nsSNPs based on the sequencing of a subset of heterozygous novel nsSNPs and the fraction of rare homozygous SNPs with known allele frequencies (see Methods). Using this approach, we estimate ∼1,600–2,000 rare nsSNPs in this individual's genome, the lower bound takes into account the ∼25% false positive rate for novel SNPs (see Methods). We conclude that ∼15–20% of the nsSNPs in an individual are rare, and ∼95% of the rare nsSNPs are heterozygous (see Methods). The number of rare variants found in this individual may guide our expectations when we sequence additional genomes in the future.

### nsSNPs Likely to Affect Protein Function Tend to be Heterozygous, Rare, or Novel

We wanted to identify the nsSNPs that may affect protein function and possibly be involved in human health and undergoing negative selection. Algorithms exist that predict whether an amino acid substitution affects protein function based on sequence conservation and/or structure [27]–[34]. When applied to human nsSNPs from re-sequencing projects, 0–30% of nsSNPs are predicted to affect function [9], [31]–[33],[35]. This range is based on datasets containing a relatively small number of nsSNPs (∼50–600) in a small number of genes (∼100–200).

What distinguishes our analysis from previous reports [9], [31]–[33],[35] is that we examine a single individual, rather than a population of individuals – thus we are establishing a benchmark for individualized genomics, as opposed to population genetics. Furthermore, we study all genes, unlike the previous studies that focused on certain classes of genes that were selected for their possible relevance in human health. For our study, we use the algorithm, SIFT (Sorting Intolerant From Tolerant) to determine if a nsSNP may affect protein function [33]. SIFT takes into account whether the amino acid change resulting from a nsSNP lies in the conserved region of the protein and the type of physiochemical change, and outputs a prediction to whether a nsSNP may affect protein function. We note that SIFT and other amino acid substitution prediction algorithms [27]–[34] only predict whether a nsSNP affects protein function. These algorithms do not predict whether a variant alters the processing or stability of transcripts.

**Figure 2:**
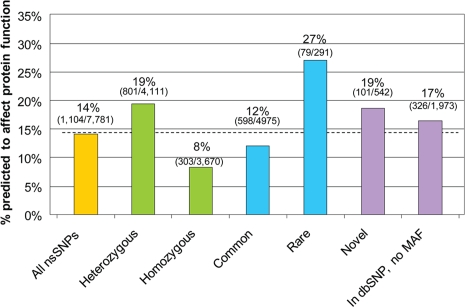
The percentage of nsSNPs predicted to affect protein function, by category. A higher fraction of heterozygous, novel, and rare nsSNPs are predicted to affect function compared to homozygous and common nsSNPs. Rare nsSNPs have allele frequencies <0.05; common nsSNPs have allele frequencies > = 0.05.

Approximately 75% of the HuRef nsSNPs had SIFT predictions (see Methods), and 14% were predicted to impact protein function (Figure 2). This suggests that the majority of nsSNPs in this individual are functionally neutral. It also indicates that an individual has ∼1,500 (14% of 10,389) nsSNPs that affect protein function with deleterious effects, and we are able to confirm a previous estimate [34]. This previous estimate was obtained by taking the average nonsynonymous nucleotide substitution rate (based on a small number of genes) and extrapolating it for all genes. Meanwhile our estimate is based on the actual number of observed nsSNPs in an individual.

The ∼1,500 nsSNPs predicted to affect protein function are deleterious in the evolutionary sense with each nsSNP having a selection coefficient *s*≈10^−3^, on average [34]. A small selection coefficient suggests that the deleterious nsSNP either has negligible effects on health, has effects late in life after reproduction has occurred, or causes a disadvantage in certain environments. We term the nsSNPs predicted to affect protein function as predicted-protein-affecting nsSNPs. The other ∼9,000 nsSNPs are effectively neutral mutations with 0<*s*<10^−4^, assuming an effective population size of 10,000 [36]. Thus, the effects of nsSNPs span a spectrum ranging from neutral to mildly deleterious, and the predicted-protein-affecting nsSNPs tend to be more detrimental.

Heterozygous nsSNPs are two times more likely to be predicted as protein-affecting compared to the homozygous nsSNPs (p<0.001; Figure 2). We reason that predicted-protein-affecting nsSNPs are expected to be selected against and therefore, less likely to both reach high allele frequencies and be observed in homozygote form. Rare nsSNPs are also two times more likely to be predicted as protein-affecting compared to common nsSNPs (p<0.001; Figure 2) and this trend has been reported previously [9],[10],[35]. This suggests that a higher proportion of the rare nsSNPs are deleterious, and are more likely undergoing negative selection. Also, a higher percentage of novel nsSNPs and nsSNPs with unknown allele frequencies are predicted to be protein affecting compared to all the nsSNPs (Figure 2), but this difference only holds for the heterozygous SNPs and not the homozygous nsSNPs (Figure S1; Text S1).

Therefore, novel, rare, and heterozygous nsSNPs are more likely to affect protein function and cause phenotypic effects. Yet rare and novel nsSNPs are difficult to characterize because they are underpowered in whole-genome association studies [4]. Thus, one of the major challenges in the future of genomics is how to correlate rare and novel nsSNPs with phenotypes.

### SNPs that Cause Premature Termination Codons

There are 105 HuRef SNPs that result in **p**remature **t**ermination **c**odons, or stop codons, in 103 genes, hereafter referred to as PTC-SNPs. This corresponds to 0.5% of coding SNPs. These SNPs are expected to result in the loss of their respective proteins and hence be under strong negative selection. Yet when we retrieved allele frequencies for PTC-SNPs, all of the 36 PTC-SNPs with known allele frequencies were common, which shows that not all PTC-SNPs are under strong purifying selection.

We investigate possible reasons for why some PTC-SNPs are not under strong negative selection and are able to reach high allele frequencies in the human population. Thirty percent (31/105) of the PTC-SNPs occur in segmental duplications in the human genome compared to 9.8% for synonymous SNPs. We assume gene redundancy would rescue these mutations, although loss of one copy of a gene can still have quantitative effects [37]. It is also possible that these PTC-SNPs have been mistakenly mapped due to the difficulty of assembling highly duplicated regions.

We remove the PTC-SNPs in segmentally duplicated regions from consideration and 74 PTC-SNPs in 73 genes remain. A substantial fraction (42%) of the remaining genes with PTC-SNPs are hypothetical. Hypothetical genes containing common PTC-SNPs may not be important to the human population, and using these variants may improve annotation of the human genome. If the PTC-SNPs in hypothetical genes are removed from consideration, 43 PTC-SNPs in 42 genes remain, and we sought to characterize these further.

Because we saw that three times as many PTC-SNPs occur in segmental duplications than expected, we postulated that multiple copies of a gene may permit the existence of a PTC-SNP. We examined the size of the gene family for the remaining 42 genes, and found that the median gene family size is 6, which is higher than the median gene family size for all genes, which is 2 (p<0.001). Thus, PTC-SNPs tend to occur in genes that have other homologues present in the genome, which may rescue the full or partial loss of a related gene [38]. There are only 9 PTC-SNPs in 8 genes that are non-hypothetical and unique members of their gene family.

In general, PTC-SNPs tend to occur in hypothetical proteins, segmentally duplicated regions, and gene families with multiple members. In the future, we may be able to use these trends to prioritize which PTC-SNPs are most likely to have functional consequences. All the PTC SNPs in HuRef can be found in Table S1, and none are in genes known to be involved in disease.

### Coding Indels

Indels are the second most abundant type of genetic variation, following single nucleotide substitutions and account for almost a quarter of the genetic variation implicated in disease [2]. Coding indels can significantly impact their corresponding genes if they introduce frameshifts that lead to unfinished protein products.

The HuRef genome contains a total of 739 coding indels, which consists of 281 heterozygous indels and 458 homozygous indels. To the best of our knowledge, this is the largest set of human coding indel variants identified to date [39].

**Figure 3:**
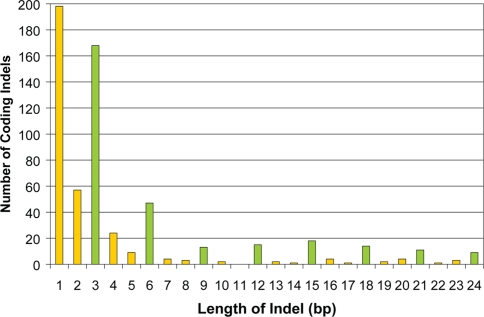
The size distribution of coding indels. Coding indels are predominantly the size of 3n, where n is an integer. 3n coding indels do not cause frameshifts, whereas non-3n coding indels do.

We find an enrichment of indels that have sizes that are multiples of 3 in the HuRef coding indel set (Figure 3). We will refer to indels that have lengths divisible by 3 as 3n indels and indels with lengths not divisible by 3 as non-3n indels, where n is an integer. In coding regions, a non-3n indel would cause a frameshift that usually leads to a truncated protein product whereas a 3n indel would cause deletion or insertion of amino acid(s). By comparing the diversity rates between coding indels and indels genome-wide (Figure S2), we find that 94% of non-3n coding indels have been eliminated by natural selection. In contrast, only 46% of 3n coding indels have been eliminated. This signifies that 3n coding indels are not as strongly selected against as non-3n indels.

Many of the indels are due to polymorphism in tandem repeat sequence. Only 6% of coding regions are classified as tandem repeats, yet 52% (381/739) of all coding indels occur in tandem repeats. The majority (73%) of the tandem repeats in coding regions have a periodicity of 3 and account for 66% (252/384) of the 3n bp coding indels. This suggests that local regions in a protein coding sequence can be prone to mutation that will either remove or insert amino acids into the protein product.

In contrast to SNPs, many indels are not validated and their allele frequencies are unknown due to difficulty in their ascertainment using either sequencing or genotyping technologies [39],[40]. To validate these indels, we verified if the indel was confirmed in the chimpanzee genome sequence [41]. We determined that 24% (181/739) of the coding indels correspond to the chimpanzee sequence. These indels are likely to be common in the human population if the indels occurred before the divergence of chimpanzee and human, although an alternative possibility is that some of these mutations are recurring events. This signifies that at least 24% of the HuRef coding indels are real.

We sought explanations of how an individual human genome could have 739 indels affecting 607 genes, and yet the individual appears to lack severe phenotypic effects. Small coding indels (< = 30 bp) account for 84% (621/739) of the indels. In the following section, we analyze the 621 small coding indels (< = 30 bp). Large indels are discussed in a later section.

### Coding Indels Are Not Randomly Located in Their Proteins

**Figure 4:**
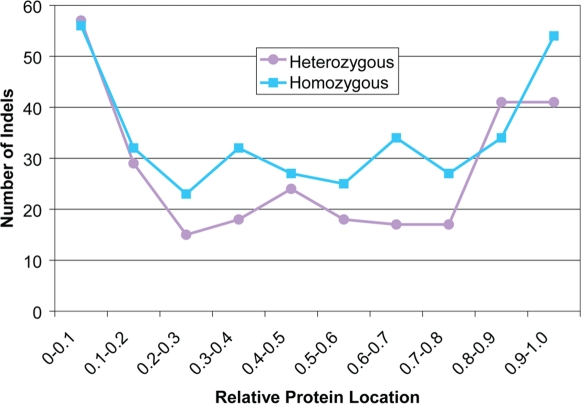
Location of coding indels. On the x-axis is the relative protein location of the coding indel, which is the first amino acid position of the indel divided by the protein length. A relative protein location near zero indicates that the indel is located near the N-terminus of the protein and a relative protein location near one indicates that the indel is located near the C-terminus of the protein. Indels occur frequently at the N- and C-termini of proteins.

Many of the small coding indels were located at the N- and C-termini of their respective proteins. We calculated the relative position of the indel in the protein by dividing the indel's position by the total protein length. With this metric, one would expect that the indel's position would be uniformly distributed across the protein. Instead, indels tend to occur at the N- and C-termini of their proteins (Figure 4). If a coding indel occurs at the C-terminus of the protein, it may not affect the function of the protein because most of the protein product has been translated successfully. If a coding indel occurs at the N-terminus of the protein, this may be rescued by a downstream start codon in the coding region (see Figure S3 for an example). This suggests that indels at the N- and C-termini of their proteins are functionally neutral and a future study using these indels to propose alternate start and stop sites could improve human gene annotation.

**Figure 5:**
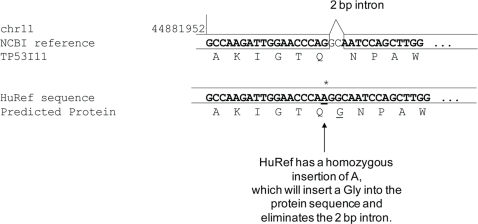
An example of a homozygous indel located near an exon boundary. The HuRef assembly has a homozygous insertion of A at chr11: 44881936. This insertion resides inside a coding exon of the gene TP53I11, but is near a 2 bp intron. With this new base inserted, a single amino acid is introduced into the protein sequence, which is the more likely scenario instead of a 2 bp intron.

Furthermore, a high proportion of homozygous coding indels were located near an exon boundary. A large proportion of the small homozygous coding indels were within 10 bp of the exon boundary: 27% (101/344) compared to 12% (34/277) for small heterozygous coding indels. Close inspection of the homozygous indels near exon boundaries showed that these indels were near small introns and the HuRef allele corrects the NCBI reference genome to provide a better gene model. Figure 5 shows an example where a 1-bp homozygous coding insertion borders a 2 bp intron, so rather than causing a frameshift, the small intron is replaced by an amino acid. Incorporating the homozygous indel from HuRef likely produces the correct protein sequence. Therefore, it is very likely that the HuRef assembly has the correct sequence, and could potentially be used to correct gene structures that were based on the NCBI human genome. Many of these small homozygous indels within 10 bp of an exon boundary were also found in chimp (61% (62/101)), further evidence that these indels are the ancestral alleles and likely to be accurate.

We re-sequenced seven homozygous non-3n indels that were either near exon boundaries (4), and/or confirmed by chimp (4), and/or at the N-terminus of a protein (1) (see Methods). These non-3n indels would supposedly cause frameshifts, yet all seven indels were found to be common (MAF>0.05) and four were determined to have an allele frequency of 1 (Table S2). This indicates that NCBI has a rare or erroneous allele at these positions and suggests that homozygous indels located at certain locations may correct the NCBI genome sequence.

We assume that indels near protein termini and near exon boundaries are functionally neutral. Removing these indels from the indel set reduces the entire set by 45%, with 342 indels remaining. Of the remaining indels, the fraction of indels that have length 3n increases from 49% (303/621) to 60% (205/342). This suggests that while the termini of a protein may be able to tolerate the presence of non-3n bp indels, elsewhere in a protein there is a greater preference for 3n indels.

We categorize the remaining indels by their 3n and non-3n lengths. Whereas 35% (71/205) of 3n indels are found in hypothetical proteins, 56% (77/137) of non-3n indels are found in hypothetical proteins (p<0.001). This suggests that non-3n indels occur in genes that can tolerate deleterious mutations, which may be pseudogenes or genes under weak selective constraints and we may be able to use these variants to identify genes that are likely not important for human health. We also noticed that the 3n coding indels that are not in tandem repeats tend to avoid regions of the protein that are highly conserved (Figure S4).

In summary, many of the indels are located at exon boundaries or protein termini and these are likely to be functionally neutral. The remaining coding indels typically have sizes of 3n, and those that do not tend to occur in hypothetical proteins. To the best of our knowledge, these trends have not been previously reported, and this suggests that a substantial fraction of coding indels are functionally neutral. In the future, we can use the HuRef indels to improve gene annotation and our observations to develop a method that distinguishes between functional and neutral indels.

### Genes with Missing or Partial Exons

We sought to understand genes missing large regions of coding sequence, leading to a gene and/or exon deletion that would render the gene non-functional. Therefore, we focused on deletions of coding sequence in this analysis. We will discuss newly observed genes and insertions of coding sequence in existing genes in a future manuscript.

We identified 1,454 exons in 1,046 genes where at least half of the coding exon's sequence was missing from the HuRef assembly. Further investigation showed that genes with “missing” exons are most likely due to low coverage or assembly issues with repetitive regions rather than the human individual truly missing part of a gene (see Methods and Text S1; Figure S5). This was confirmed by resequencing a subset of the missing exons in the HuRef sample and validating that in fact, most of the “missing exons” are present in the HuRef sample (see Methods and Text S1).

### Variants in Disease Genes and their Associated Phenotypes

We examined the HuRef variants in genes known to be involved in disease (based on the OMIM database [42]) to make correlations between the HuRef variation and possible phenotypes. There are a total of 682 nsSNPs in 443 disease genes (Table S3). The allele frequency distribution and fraction of predicted-protein affecting SNPs are similar to non-disease genes (Figure S6).

We examined the nsSNPs from dbSNP [43] that were found in the disease database OMIM [42] (see Text S1). Using this approach, seven HuRef nsSNPs were found to be associated with disease (Table 2). The HuRef individual is heterozygous for all seven SNPs and most of these disease-associated SNPs are common in the population. It may be considered surprising that these nsSNPs are common since they were found in the OMIM disease database. This is due to the fact that we looked for overlap with nsSNPs that are in both OMIM and dbSNP, and dbSNP tends to be biased for common SNPs [21].

**Table 2:** HuRef nsSNPs with known disease associations.

SNP^a^	Gene	Genotype-Phenotype^b^
rs6265	BDNF	MAF = 0.18
V74M		Increased risk for eating disorder OR = 1.6 [84]
		Met/Met: Inferior episodic memory [85]
		Met/Met: Later onset of Parkinson's disease [86]
		Met: In bipolar patients, less adaptive to change [87]
		Predicted to affect protein function.^d^
rs1800556	ACADS	MAF = 0.17
R171W		ACADS with 171W has residual activity (45%). Because this is polymorphic in the control population, this is a predisposition allele that can cause SCAD deficiency if additional factors are present [88].
		Predicted to affect protein function.^c^
rs1805389	LIG4	MAF = 0.02; MAF = 0.07 in [89]
A3V		1.5-fold reduction in risk of developing multiple myeloma for heterozygotes [89].
		Predicted to be functionally neutral.^d^
rs13073139	BTD	MAF = 0.17 [90]
A171T		Biotinidase deficiency, asymptomatic in heterozygous form [91]. A171T is in linkage disequilibrium with D444H and D444H results in 48% of normal enzyme activity. We confirmed that HuRef is heterozygous for D444H. It is unknown whether A171T produces nonfunctional enzyme.
		A171T is predicted to be functionally neutral.
		D444H is predicted to affect protein function.^c^
rs2303067	SPINK5	MAF = 0.48. Associated with allergies, atopic dermatitis, asthma, and total serum IgE. Paternally derived alleles tended to be less often associated with disease than maternal alleles [92]
E422K		Predicted to be functionally neutral.^d^
rs11556045	HEXB	MAF = 0.22. Observed in a patient with juvenile Sandhoff disease, but the patient had another mutation which activated a cryptic splice site. This SNP is unlikely to be the causative variant. [93]
K121R		Predicted to be functionally neutral.
rs4880	SOD2	MAF = 0.44. Risk of prostate cancer depends on genotype, vitamin E uptake, and smoking status. For the heterozygote, there is an increased risk of prostate cancer. Val/Val: OR = 1. Val/Ala OR = 1.17 Ala/Ala = 1.28
A16V		This risk is increased with smoking and low vitamin E uptake [94].
		Within hereditary haemochromatosis patients, carriers of the Val allele have a higher prevalence of cardiomyopathy [95].
		Mutant protein has 30–40% lower activity.
		Predicted to be functionally neutral.^c^

a All of these SNPs were heterozygous in HuRef.

b OR = odds ratio. We note that these associations should be interpreted with caution because there are disagreements in the published literature [94], [96]–[98]. Additional genotype-phenotype relationships for this individual can also be found in Table 13 of [16].

c Three nsSNPs have been shown to cause reduced protein activity. Of these, two are predicted to affect function.

d Three nsSNPs associated with disease but for which enzymatic assays have not yet been carried out (to the best of our knowledge) and are presumed to be involved in disease. It is possible that these nsSNPs are not the etiological variants but instead they could be in linkage disequilibrium with the etiological variants. One of these three nsSNPs is predicted to affect protein function.

From these seven variants, one could simplistically infer that the HuRef individual has an increased risk for eating disorder (BDNF), 1.5-fold reduced risk to multiple myeloma (LIG4), an increased risk to prostate cancer which can be rescued by taking vitamin E supplements (SOD2), and allergic tendencies (SPINK5) (Table 2). Some of the SNPs have known interactions with environmental factors (SOD2 and BDNF in Table 2). However, the published risks for these variants are from population-based studies, and may not apply to this specific individual because it does not take into account other interacting genetic loci and his environment [44]–[47]. Therefore, Table 2 does not show exact risks for this individual and predicting the phenotype or lack of phenotype of this individual is premature. These variants, like many of the risk variants being uncovered by genome-wide association studies, have low risks and we may not have a clear understanding of their clinical utility until all the relevant factors (both genetic and environmental) for a particular disease have been elucidated [44]–[47].

These seven well-studied examples demonstrate the complexity of trying to interpret variants and their impact, but there are still many variants in this individual that are uncharacterized. For the 682 nsSNPs in 443 disease genes, 27 are rare (MAF<0.05), 18 are novel, and 81 are predicted to affect function (Table S3). Interpretation of these variants is difficult because of the absence of literature for many of the observed variants. One challenge is that these variants, even if they affect protein function, could be phenotypically neutral in certain contexts (see SOD2 and BDNF in Table 2 and [48]). Also, even if there is evidence that a nsSNP is under negative selection (e.g. predicted to affect function and/or rare), it is not straightforward to interpret a possible phenotype because mutations at different locations in the same gene can have different effects [49]. The difficulty of inferring phenotypic consequences from a variant is depicted in the following example. rs562556, which is homozygous in HuRef and has an unknown minor allele frequency, introduces the amino acid substitution V474I in PCSK9, and this amino acid substitution is predicted to affect protein function. Because defects in PCSK9 cause familial hypercholesterolemia (OMIM∶607786), one could speculate that this SNP could affect the donor's cholesterol levels. However, extensive functional studies of this variant and others are necessary before any conclusions can be made. Because only 1% (7/682) of the nsSNPs in disease genes in this individual human have been well-characterized, this indicates that we are only at the beginning of relating genotypes to phenotypes, even for the well-characterized disease genes.

There were 28 indels in 26 disease genes (Table S4). Only 3 out of the 28 indels have lengths that are not multiples of 3n, and would cause frameshifts. Two of these indels appear to be annotation issues and are likely to be functionally neutral (see Text S1). The third indel is in ACOX2. This protein is involved in lipid metabolism, and patients with Zellweger syndrome lack this protein [50]. Because the indel is heterozygous in the HuRef individual and one functional copy is present, the individual may not be adversely affected by this mutation.

For the remaining 25 indels with length 3n in 23 disease genes, 84% were in tandem repeats. Some of these genes are known to cause disease due to polyglutamine and polyalanine repeat expansions (AR, ATXN2, ATXN3, HD, TBP). For these genes, we confirmed that the number of repeats in the HuRef genome falls within the range of what is observed for unaffected individuals. In addition, this individual is heterozygous for a 24-bp duplication in the CHIT1 gene, which activates a cryptic 3′ splice site and causes chitinase deficiency [51]. Even though the indel produces a nonfunctional protein, the indel is observed at an allele frequency of 23% in the general population. One possibility for its high incidence is that this indel may provide a selective advantage against fungal pathogens [51].

### Selective Constraints on the Human Genome

To examine selective constraints on different regions across the genome, we estimated the diversity rate θ (see Methods). We calculate the diversity rates for regions throughout the entire genome as well as calculating the diversity values for genic regions (Table 3). Values of θ tend to be negatively correlated with the strength of selection, where low values of θ tend to indicate strong selective pressures, while high values do not. The order of θ is: coding in disease genes<coding<conserved noncoding intronic<splice sites<3′UTR≈5′UTR≈conserved noncoding intergenic<promoter<introns<repeats. This indicates that coding regions in disease regions are the most selectively constrained regions and repeats are the least. Some diversity values and trends have been reported in previous publications [6]–[8],[52],[53], and our results are in agreement with these values. However, to our knowledge this is the first study with such an extensive list of regions. We also find indels are significantly under-represented in coding regions; there is a 43∶1 SNP∶indel ratio for coding regions compared to a 7∶1 SNP∶indel ratio genome-wide. It is reasonable to observe stronger selection against indels in coding regions where they can introduce frameshifts.

**Table 3:** Diversity Rates for Autosomal Chromosomes.

	SNP Diversity (×10^−4^)^a^ (based on Dr. Venter's genome)	Indel Diversity (×10^−4^)^b^ (based on Dr. Venter's genome)	SNP Diversity (×10^−4^)^c^ (based on Dr. Watson's genome)
Total	6.2	0.9	6.5
**Gene Regions**			
CDS	3.6	0.08 Filtered: 0.09	4.0
CDS of disease genes	2.9	0.06 Filtered: 0.04	3.0
Constitutive exons	3.5	0.08 Filtered: 0.08	4.0
Alternative exons	4.6	0.1 Filtered: 0.06	4.8
5′UTR	4.4	0.3	4.9
3′UTR	4.7	0.7	5.1
Splice sites	4.0	0.6	4.6
Promoter ( 1 kb upstream)	5.4	0.8	6.1
Introns	5.6	0.9	6.2
**Conserved Elements**			
All	4.3	0.5	4.9
Intronic conserved	3.8	0.5	4.3
Intergenic conserved	4.7	0.5	5.2
**Repeats**			
All	6.6	1.2	7.5
Alu	7.2	2.6	7.5
MIR	5.6	0.3	6.1
MER	6.3	0.5	7.0
LTR	7.2	0.4	8.0
L1	6.6	0.6	7.2
L2	5.6	0.4	6.3
Simple repeats (xxx)n	8.8	15	19

a The diversity rates based on Dr. Venter's and Dr. Watson's genomes are probably underestimated by ∼25%, the percentage of heterozygotes missed due to low read coverage [16],[20].

b For coding indels, we show the diversity values before and after filtering. Some diversity values for indels are higher after filtering because homozygous indels were re-classified as heterozygous indels.

c For Dr. Watson's genome, we assume that the entire genome was covered by reads, which will also lead to an underestimate (see Methods). There is a large difference between the diversity values for Dr. Watson's and Dr. Venter's genome for simple repeats. This may be due to the methodological differences between Sanger and 454 technologies.

We further explored if the observed HuRef variation could indicate whether certain genes were under stronger selective constraints than others. We identified 538 genes containing common nsSNPs located in conserved regions of the protein and are predicted to affect protein function. We called this set of genes the Commonly-Affected genes. In parallel, we identified 79 genes containing rare nsSNPs predicted to affect protein function, and we termed this set the Rarely-Affected genes.

**Figure 6:**
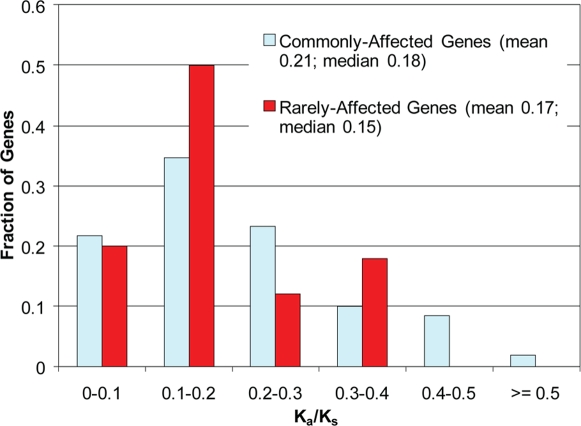
The K_a_/K_s_ ratios of Commonly-Affected genes and Rarely-Affected Genes. Commonly-Affected genes have a higher K_a_/K_s_ ratio than Rarely-Affected genes, which suggests that Commonly-Affected genes are under weaker selection.

We hypothesized that Commonly-Affected genes would be under weaker selective constraints since predicted-protein-affecting SNPs, common in the human population, were found in these genes. K_a_/K_s_, is a metric used to quantify selection of a gene and is calculated as the ratio of the nonsynonymous (amino-acid affecting) substitution rate to the synonymous substitution rate. A high K_a_/K_s_ ratio indicates that a gene is undergoing weak selection, although positive selection is also a possibility if K_a_/K_s_ is >1 across the entire gene or part of the gene [54]. We observe that the Commonly-Affected genes tend to have higher K_a_/K_s_ ratios than the Rarely-Affected genes (p = 0.09; Figure 6). This suggests that Commonly-Affected genes may not be under strong selective constraints. Presumably, mutations affecting gene function in the Commonly-Affected genes will not have significant consequences on human health, which is why these predicted-protein-affecting nsSNPs can rise to high allele frequencies. In support of this hypothesis, the Commonly-Affected genes are dominated by olfactory receptors (78/538) which is consistent with the previous observation that humans do not depend on olfaction to the same extent as other species [55].

In the future, extending this type of analysis to nsSNPs from HapMap or additional human genomes may allow identification of additional Commonly-Affected genes. This could help improve the scientific community's knowledge of the human genome by identifying which genes may not play an important role in human health if mutated.

### Comparing Individual Genomes

We also compared the genome properties of Dr. James Watson [20] to those of Dr. Craig Venter. SNP diversity rates based on Dr. Watson's genome are slightly elevated compared to Dr. Craig Venter's (Table 3), but in general, follow a similar trend as stated previously. The one exception is simple repeats, which have a 2-fold higher SNP diversity rate in Dr. Watson compared to Dr. Venter; this may be due to differences between sequencing technologies.

Of the 3.1 million SNPs in Dr. Venter's genome that map to the NCBI genome, 56% are shared with Dr. Watson. There are 606,719 and the 288,723 novel SNPs that map to the NCBI human genome in Dr. Watson's and Dr. Venter's genomes, respectively. One possible reason for the smaller number of novel SNPs in Dr. Venter's genome is because Dr. Venter's genome is partially represented in the Celera human genome assembly [16],[56], and variants have been mined from the Celera assembly and subsequently deposited in dbSNP [57]. Of the novel SNPs, only 32,528 SNPs are shared between the two individuals. This demonstrates the value of sequencing additional human genomes to discover novel variants.

We compare the number of nsSNPs and coding indels between the two exomes (Table 4). Similar numbers of nonsynonymous variants are detected in both individuals. However, 14% and 20% of nsSNPs are predicted to affect function in Dr. Venter's and Dr. Watson's exomes, respectively. One possible reason for this difference could reside in the different prediction algorithms employed [20],[30],[33]. Future analyses that use a standardized approach may clarify this apparent difference. The number of indels in Dr. Watson's genome is less than half of the number of indels we observe in HuRef (Table 4), most likely because 1 bp indels were discarded [20]. As more individuals are sequenced, scientists will be able to establish general trends and the average values for metrics that characterize a human individual's genome.

**Table 4:** Characterization of Dr. Venter's and Dr. Watson's exomes. Numbers for Dr. Watson's exome are taken from [20].

	Dr. Venter's Exome	Dr. Watson's Exome
Total Number of Nonsynonymous SNPs	10,389	10,569
Number of Novel Nonsynonymous SNPs	772 (7% of total nsSNPs)	1,573 (15% of total nsSNPs)
% nsSNPs predicted to affect protein function*	14% (7,781 predicted on)	20% (3,898 predicted on)
Number of Coding Indels	739	345**

*: Different prediction algorithms were used [30],[33], and this may account for the difference between the two exomes.

**: Indels of size 2 bp and greater were considered; 1 bp indels were discarded. If we removed 1 bp indels from Dr. Venter's exome in order to compare with Dr. Watson's exome, Dr. Venter would have 423 coding indels.

## Discussion

Coding exons are believed to be rich in functional variation because many coding mutations have been found to cause phenotypic effects [58]. As a result, re-sequencing projects tend to focus on coding regions [5]–[11], multiple technologies that specifically target the exome are being developed [13]–[15],[58], and the 1000 Genomes Project may specifically target the exome [12]. This motivated us to focus on the variation in an individual's exome to see what insights could be gleaned. Protein-coding exons are thought to amount to a little less than half of the functional portion of the genome; noncoding highly conserved regions constitute the other half [59]–[62]. We did not analyze noncoding variants even though they can be involved in disease [3], [63]–[66] because exons are the best characterized regions that correlate to phenotypes and it is difficult to characterize the impact of noncoding variants at this time. As our understanding of non-genic regions increases, so can we expand our interpretation since the data for this individual's entire genome is available.

### Personalized Genomics Today

This is the first study of an individual's exome and we establish what one may expect to observe from the variation in the exome of an individual. We show that the majority of coding variants in a human are neutral or nearly neutral. This is not unexpected, since we know that this genome creates an individual who has survived past 60 years of age. We also find that within an individual, the basic principles of genetics are followed. Additionally, we examined the variation in genes known to be involved in disease, and found no indication that the individual should have a severe disease, which matches the phenotype currently known.

Despite having a human's complete genome sequence, we are only at the tip of the iceberg for understanding how an individual's genotype and phenotype are related. One significant challenge is that the phenotypic effects of the majority of genes are unknown. Currently, only 7% of genes are annotated with OMIM disease associations so that it is difficult to predict the phenotypic effects of variants for a large proportion of genes. If one is to rank genes by importance and effect on phenotype, then based on the results of this study, one might consider that genes containing PTC-SNPs, frameshifting indels, and damaging nsSNPs that are common in the human population to be under weak selection, and variants in these genes may not be as relevant to human health. Several groups have used gene ontology, literature, and other sources to predict potential disease genes [67],[68], we propose that one can also use observed human variation to increase our understanding of the human genome.

Even if a gene is known to be involved in disease, it is difficult to understand if a variant in the gene will have a phenotypic effect. We found that 99% of the nsSNPs in disease genes could not be characterized by current literature. Different mutations in the same gene can cause different phenotypic effects [49], thus making it difficult to interpret possible phenotypes. Furthermore, some variants have phenotypic effects only under certain environments (see SOD2 and BDNF in Table 2 and [48]). Also, when looking at complex phenotypes, multiple variants in coding and non-coding regions are likely to be involved [63]–[66]. This genetic complexity, as well as exposure to various environmental factors, will need to be taken into account in assessing risk for various diseases.

How can geneticists start to grasp the significance of phenotype-genotype correlations? This question is especially relevant to companies offering personalized genomics to their consumers (e.g. 23andMe, Navigenics, deCODE Genetics). When looking amongst the human population, there are many rare SNPs, but when looking at a single human individual, the majority of the SNPs are common [6],[69],[70], and in this study we estimate that >80% of the nsSNPs in an individual are common. Therefore, understanding which common variants are involved in common disease will greatly benefit an individual, because common variants account for a significant fraction of the variation in each human. Recent genome-wide association studies have identified common variants implicated in disease [71]–[73] and these studies will continue to find common disease-associated variants in the near future. These discoveries will be valuable for interpreting a large proportion of an individual's genome. However, one should be cautious in the interpretation of these variants because variants with low associated risks may not necessarily have good predictability in the clinical setting [44],[45].

In contrast, rare variants are harder to study because genome-wide association studies are insufficiently powered to detect rare variants [4]. We found that a higher fraction of rare nsSNPs were predicted to affect protein function compared to common nsSNPs, in agreement with previous studies [9],[10],[35]. This suggests that a small proportion of a large number of common variants and a larger proportion of a small number of rare variants will contribute to the health of a human individual. Genome-wide association studies tend not to have the power to detect rare etiological variants [4] so that predicting whether a rare mutation in an individual causes disease without any other phenotypic information is extremely difficult. Therefore, one of the major future challenges in personalized genomics is the interpretation of the effects of rare variants found in an individual, especially if this information will be relayed back to the individual and could impact the person's lifestyle.

In addition to interpretation and analysis, much effort was expended in ensuring that a variant was authentic (see Methods). There could be unintended negative consequences for telling a person that they have a disease variant, when in actuality the variant could be detected in error. Common reasons for our false positive variant calls were technical sequencing error, low sequence coverage, and low-complexity sequence. When interpreting an individual's genome that can potentially impact a person's lifestyle, manual curation, editing, and verification by other technologies seems prudent.

### Improving the Currently Available Reference Human Genome Sequence

The human genome sequence has had a significant impact on research since its availability in 2001 [56],[74], but our analysis suggests that rare or erroneous alleles may have been incorporated into the NCBI human genome sequence, and that this sequence can be corrected and improved. Because one of the goals of the 1000 Genomes Project is to improve the human reference sequence [75], our study points to where such improvements can be made. We find that ∼80% of the homozygous SNPs in HuRef tend to be the major allele in the population (Figure 1), and ∼20% of the homozygous HuRef nsSNPs had allele frequencies equal to 1. Thus, at the majority of HuRef homozygous positions, the NCBI human reference sequence has the minor allele. If the scientific community sequenced many individuals, it could determine the major allele at each position in the genome. If the major allele was incorporated into the coding sequence, and this was used in subsequent gene prediction models, then the predominant form of the protein in the human population would be represented instead of a rarer form. Also, by using the common allele instead of the sometimes rarer NCBI allele, the number of perceived variation would be reduced when comparing human genomes. For example, if the NCBI human genome sequence were to incorporate the major allele and HuRef was then compared to this modified NCBI genome sequence, then we estimate the new number of HuRef homozygous SNPs genome-wide would be ∼300,000 (0.2 * 1.45 million [16]) and the number of total variants between HuRef and the modified NCBI would reduce from 4.1 million [16] to 3 million. Similarly, if we remove the homozygous nsSNPs that correspond to the common allele in European population (AF>0.5), then the fraction of rare nsSNPs increases by 6%. This demonstrates the importance of the human genome reference sequence for the evaluation of variation in individual human genomes.

The scientific community could also make use of coding indel variation to correct and improve gene annotation. Indels near exon boundaries appear to provide the correct gene models to give the appropriate protein product (Figure 5, for example) [76]. Gene annotation for translation starts and stops can be further refined based on our observation that coding indels are found frequently at the N- or C-termini of their proteins. Frameshifting-indels at the N-termini of proteins could indicate that a translation start site further downstream may be the true start codon, or at least an alternative start codon can still yield a functional protein. Indels such as these may be polymorphic and so accounting for these indels could simplify future analyses on exomes as they could be quickly regarded as functionally neutral and reduce the total number of indels that need to be analyzed.

We also observed a trend where common predicted-protein-affecting nsSNPs, PTC-SNPs and frameshift-inducing indels tend to occur in hypothetical genes. This suggests that these genes are not under strong selective pressures and mutations in these genes may not be relevant in the human population. Future studies with more human sequences identifying additional nonfunctional mutations in genes would help us confirm whether these genes are essential.

### Sequencing of Many Human Genomes

Our exome analysis is currently limited to one individual. However, there will be significant benefits from sequencing many individuals whose phenotypes are known. One can envision collecting the genetic variation from these genomes and grouping individuals based on their respective phenotypes. Then for each phenotype, one may discover the genes which are involved in disease by looking collectively at the rare and common variants [11],[77],[78]. The analysis can also be strengthened by analyzing pathways instead of individual genes [78].

Furthermore, whole genome sequencing means we need not be limited to the exome. Using whole genomes, one could look for clustered mutations in conserved regulatory elements, especially since many association studies have found disease-associated loci in non-coding regions [63]–[66]. To assess the role of noncoding variation, we examined HuRef variation in and around genes involved in the melanoma pathway because the HuRef donor has reported a case of melanoma (Figure S7). We found that the majority of the variants occurred in conserved noncoding regions. This suggests that it may be insufficient to sequence just the exome and it is important to understand all types of variation, coding and noncoding, as well as interactions with the environment when studying phenotypes.

### Conclusion

**Figure 7:**
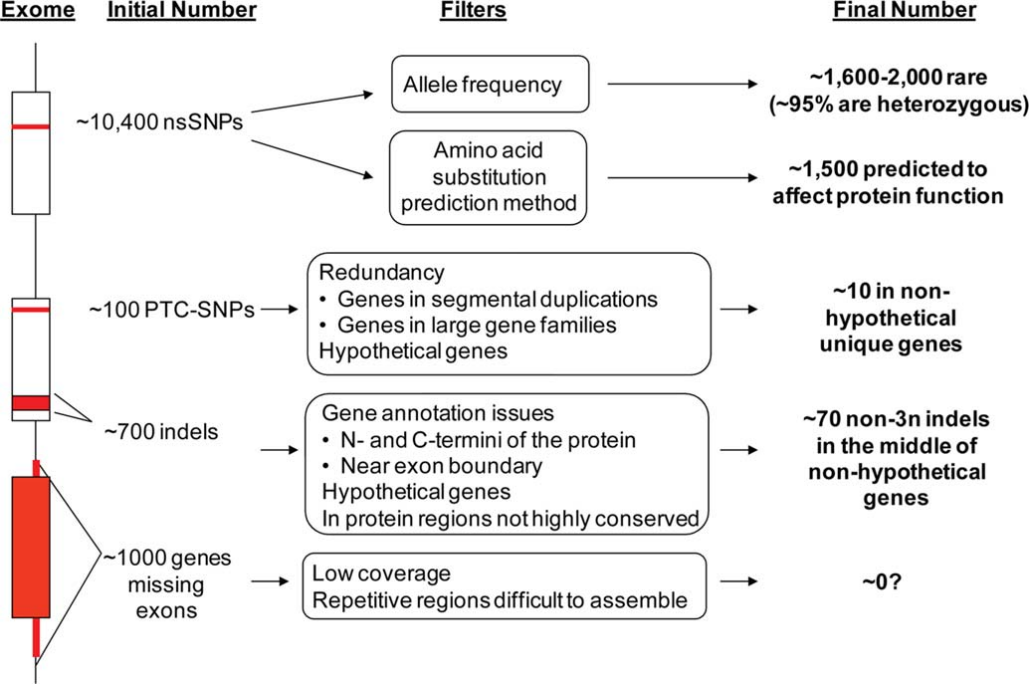
A summary of the nonsilent coding variants and their observed trends.

We have filtered the initial set of ∼12,500 coding variants that affect protein sequence to a substantially smaller set that are most likely to have major effects on gene function (Figure 7). The trends that we have detected suggest we can reduce the number of putative functional coding variants by ∼8-fold and will provide a future guide for how one can analyze coding variants when additional human genomes are sequenced in the future. Additionally, the variants found here and in future studies may be used to improve our understanding of the human genome by correcting gene annotation and identifying genes not likely to be relevant to human health. We anticipate that this study will help guide the scientific community's expectations and experimental design in future genome sequencing projects.

## Methods

### Variant Set

We used the filtered variant set as described in [16]. We define homozygous variants as loci where the alleles differ from the NCBI reference genome, but are the same within the HuRef assembly. This variant set was used to generate the diversity values in Table 3. For all other sections in this manuscript, quality inspection of variants was performed. To assure the quality of novel coding variants, we inspected manually the sequencing traces of novel heterozygous nsSNPs, all heterozygous PTC-SNPs, and all coding indels less than 20 bp in length. The sequence traces for these coding variants were extracted and three people independently reviewed the traces, by examining the quality of the traces and determining whether the variant was correctly called. If at least two people confirmed the existence of the variant, the variant was deemed acceptable, otherwise the variant was discarded. 35% (424/1196) of the novel heterozygous nsSNPs, 12% (9/73) of the heterozygous PTC-SNPs, and 33% (355/1088) of the coding indels were discarded. This may suggest that a significant fraction of the variants reported in [16] are dubious. However, this analysis is restricted to coding variation which is known to be under strong selection compared to the rest of the genome. Hence, there will be fewer real variants in coding regions and a higher proportion of the novel coding variants will be false positives. 20% (123/611) of the homozygous indels were reclassified as heterozygous because there was trace evidence for a second allele.

The importance of filtering is demonstrated with the following observation. Manual inspection reduces the number of novel nsSNPs by a third, but especially filters out a higher proportion of predicted-protein-affecting nsSNPs. Prior to filtering, the number of novel heterozygous predicted-protein-affecting nsSNPs is 195, after filtering this is more than halved to 89 novel nsSNPs predicted to affect protein function.

### SNP Characterization

SNPs not found in dbSNP v. 126 [43] were designated as novel. All allele frequencies were based on the CEU samples genotyped from the HapMap Project [24],[25], unless otherwise stated. Allele frequencies for 72% (3429/4785) of the homozygous nsSNPs and 63% (3544/5604) of the heterozygous nsSNPs were obtained. For heterozygous SNPs, we report the minor allele frequency (MAF). For homozygous SNPs, we report the CEU allele frequency of the allele observed in the HuRef genome. We define common SNPs as SNPs with allele frequencies > = 0.05 and rare SNPs with allele frequencies <0.05.

The amino acid changes resulting from coding variants were determined by SNPClassifier, an internally developed software tool. The HuRef variants, their alleles, and positions in genomic coordinates, are provided as input into SNPClassifier. Annotation is automatically retrieved from Ensembl and is used to assign variants to defined gene categories. Variants in or near genes can be subtyped as: promoter (1 kb upstream of the transcription start site), intronic, 5′ UTR, 3′UTR, coding, or downstream of the transcript (1 kb). Coding SNPs are designated either as synonymous or nonsynonymous and coding indels are designated as either frameshift or amino acid insertions/deletions. The resulting protein product from coding indels that introduce frameshifts is also output.

SIFT predictions for nonsynonymous SNPs were obtained by using SIFT 2.1.1 [33]. The protein sequences containing nonsynonymous SNPs were searched against SwissProt-Trembl 54. Confidence in predictions is measured by the median sequence information, we used a cutoff of 3.5 for confidence. Approximately 75% (7,781/ 10,389) of the nsSNPs had SIFT predictions, the remaining 25% did not have a sufficient number of homologous sequences that are needed for prediction.

### Estimating Number of Rare NsSNPs

We estimate the number of rare nsSNPs with allele frequency (AF) <0.05 in an individual. For the 67% nsSNPs with known AFs from the HapMap Project, there are 56 rare homozygous nsSNPs and 326 rare heterozygous nsSNPs. For the 1,356 homozygous nsSNPs with unknown AFs, the percentage predicted to affect function is similar to that seen for homozygous SNPs with known AFs (Figure S1). If the homozygous nsSNPs had a higher proportion rare SNPs, then a higher fraction should be predicted-protein-affecting but because they are similar, we assume that the homozygous nsSNPs with unknown AFs have a similar proportion of rare SNPs as the homozygous nsSNPs with known AFs. Because 1.6% (56/3429) of the homozygous nsSNPs with known AFs are rare, we estimate ∼22 (1.6% * 1,356) of the homozygous nsSNPs with unknown AF are rare, so in addition to the 56 rare homozygous nsSNPs with known AF, there is a total of ∼80 rare homozygous nsSNPs in this individual. For heterozygous nsSNPs, there are 326 heterozygous rare nsSNPs with known MAF, and 2,060 heterozygous nsSNPs with unknown MAF. From sequencing, as much as a quarter of the heterozygous nsSNPs with unknown MAF could be false positives, although this estimate is likely to be an upper bound (see Sequencing for Variant Validation in Methods). Therefore the range of novel heterozygous nsSNPs falls within ∼1,550–2,060. We also ascertained from sequencing that ∼75% of the heterozygous novel nsSNPs are rare. Therefore, we estimate that ∼1,200–1,550 of the heterozygous nsSNPs with unknown MAFs are rare and in total, there are ∼1,500–1,900 rare heterozygous nsSNPs. Thus, we estimate ∼1,600–2,000 rare nsSNPs in this individual's genome, and ∼95% of the rare nsSNPs are in heterozygous state.

### Indel Characterization

For an indel's location, we calculated the relative position of the indel in the protein by taking the first amino acid position affected by the indel. We divided the position by the total length of the protein, so that a relative protein position value close to 0 indicates that the indel affects the N-terminus of the protein, and a relative protein position value close to 1 indicates that the indel affects the C-terminus of the protein. We designate that an indel affects the N-terminus of a protein if the relative protein position is between 0 and 0.1; an indel affects the C-terminus of a protein if the relative protein position is between 0.9 and 1.0. Thus, an indel is said to affect the N-terminus or C-terminus of the protein if it lies within the first 10% or last 10% of the open reading frame, respectively.

To examine whether an indel occurs in a conserved region of the protein, the sequence alignment of the protein sequence with homologues from other organisms were retrieved from Ensembl. At every position in the protein alignment, sequence conservation was calculated [79]. The conservation value at the indel's position is compared with all other positions, and the percentile rank is calculated. If the number of sequences in the alignment was less than 10, the data point was removed.

### Genes Missing Exons

The HuRef assembly was mapped by an assembly-to-assembly comparison to the NCBI build 36 human reference genome [16]. Regions in NCBI reference that were missing in the human diploid assembly were identified. We intersected the missing regions with coding exons greater than 50 bp in length and ensured that at least 50% of the exon was missing from the HuRef assembly in order to consider the exon. To double-check that the missing sequence was not in unassembled sequence, we searched the exonic sequence using MEGABLAST [80] against the HuRef assembled sequence and the unassembled singletons. MEGABLAST hits greater than 95% identify and with 50 bp minimum length were kept. We decided exons were not truly missing if >90% of its length were covered by these MEGABLAST hits. The final set consisted of 1,454 exons in 1,046 genes.

We removed the genes located on sex chromosomes because the sex chromosomes are known to have low coverage [16]. After removing these genes, there were 719 genes with 880 missing or partial exons. To investigate read depth for this set of exons, we re-mapped all untrimmed reads from [16] to the set of exons using ‘snapper’ (http://kmer.wiki.sourceforge.net/), a seed-and-extend mapper. All 20-mer seeds were extended, and any alignments over 94% identity were reported. As a control, we also remapped reads to a set of exons that were randomly selected from all exons. Whereas the control exons show a normal distribution with the median number of reads centering at 7.6, the missing exons show a bimodal distribution with either very few reads or many reads (Figure S5b). This reflects that genes with “missing” exons are most likely due to assembly issues with repetitive regions or low coverage. 66% of the “missing” exons have an average read depth of less than 2 reads, which emphasizes the importance of adequate coverage in a human genome.

### Sequencing for Variant Validation

We generated PCR primers to 15 regions in 12 genes with ‘missing exons’, 9 PTC-SNPs, 15 coding indels, and 26 novel heterozygous nsSNPs (Table S2). These 65 PCR primers consistently amplified their cognate genomic regions in 46 unrelated CEU individuals and the HuRef sample. The DNA for the HuRef sample was extracted from whole blood (see Methods in [16]). We sequenced the PCR products using Sanger dideoxy sequencing (see [16] for sequencing protocol). The 46 unrelated CEU individuals were part of the HapMap CEU panel, and their Coriell identifiers are provided in Table S2.

Of the 26 heterozygous novel nsSNPs, 6 failed to be confirmed in the HuRef sample and instead matched the NCBI allele. There was also 1 nsSNP that failed to be confirmed in the HuRef sample but was observed in other samples and this was considered to be a false negative. This suggests that the false positive error for HuRef's novel nsSNPs is ∼25% (23% = 6/26). This estimate is likely to be an upper bound due to the following reason. The 26 nsSNPs occurred in non-hypothetical genes, and nsSNPs in hypothetical genes may be under little or no selective pressures compared to nsSNPs in non-hypothetical proteins and the former can reach high allele frequencies. Hence, this false positive error may be inflated. For the novel nsSNPs that we could confirm in HuRef, the mean MAF of the novel SNPs was 0.09 and 74% (14/19) of the SNPs were rare (MAF<0.05).

For the PCR products spanning missing exons, 14 regions from 11 genes were successfully amplified in the HuRef sample and this confirmed that HuRef is not missing exons for these genes (Table S2). In the 12th gene PRED58, a 66 bp coding deletion in HuRef was observed but this was seen in all other DNA samples, suggesting that NCBI has the rare or erroneous allele.

### Genome Annotation

All genome coordinates are with respect to NCBI build 36 and all gene designations are with respect to Ensembl v. 41. A gene was considered hypothetical if in its gene description, it had no description or was described as an “open reading frame”, an “orf” which signifies an open reading frame, a cDNA clone, putative, probable, uncharacterized, “similar to” another protein, a pseudogene, a fragment, hypothetical, a novel protein, novel transcript, or if it was invalid as described in Clamp et al. [81]. Under this classification, there were 20,561 non-hypothetical genes and 10,624 hypothetical genes consisting of 29,401,727 bp and 6,176,706 bp respectively. K_a_/K_s_ is the ratio of the nonsynonymous substitution rate to the synonymous substitution rate. K_a_/K_s_ values based on human-mouse orthologous gene pairs were retrieved from Ensembl Biomart. In Table 3, constitutive exons are those coding exons that are expressed in 100% of the transcripts for its given gene. If a coding exon was present in <50% of the transcripts, it was designated as an alternative exon. Splice sites include the 20 bp within exon boundaries (10 bp intronic, 10 bp exonic for each exon boundary).

Segmental duplication regions were taken from the UCSC genomicSuperDups file (>1 kb length, >90% identity). TandemRepeatFinder [82] was used to designate tandem repeats using the parameters match = 2, mismatch = 5, delta = 5, PM = 75, PI = 20, minscore = 35.

Non-genic conserved regions were taken from phastConsElements17way that were > = 50 bp in length. If any part of the PHAST region intersected with coding, 5′UTR, and/or 3′UTR, the PHAST conserved element was removed. Therefore, the conserved regions in Table 3 were not overlapping or bordering coding, 5′UTR, or 3′UTR regions.

### Sequence Diversity

We estimate diversity θ [83] as θ = K/*a*L, where 
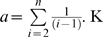
. K is the number of variants identified, L is the number of base pairs, and n is the number of alleles. For indels, K is the number of indel events. In the case of a single diploid genome, n = 2, so *a* reduces to 1. Then θ = K/L which is simply the number of heterozygous variants divided by the length sequenced. The 95% confidence interval for θ is [0, θ+2θ] or [0, 3θ], as calculated in [16].

Diversity values were calculated for the various types of regions listed in Table 3 and the counts for these values can be found in Table S5. We also attempted to look at the diversity values for gene ontology categories, but were unable to do so because of the low numbers of coding variants per gene (data not shown).

Diversity values for Dr. James Watson's genome were calculated using the 1.86 million heterozygous SNPs reported in [20]. For the denominator L, we assumed the entire chromosome was covered by reads and used the chromosome lengths from the UCSC genome browser. If this assumption is not true, then an inflated L will underestimate θ. Diversity values for indels were not calculated because indel data was not available for Dr. Watson's genome.

## Supporting Information

Figure S1 Protein-affecting predictions for nsSNPs that are novel or in dbSNP with unknown allele frequencies. When categorized by zygosity, the percentage of predicted-protein-affecting nsSNPs is similar between the different categories for homozygous nsSNPs, but not for the heterozygous nsSNPs.
(0.25 MB TIF)

Figure S2 Diversity rates for indels, based on size.
(0.12 MB TIF)

Figure S3 An example of an indel that occurs at the N-terminus of the gene MTCH1. This indel occurs in the first exon, about 30 bp after the translation start site. A start codon just downstream of the indel may serve as an alternative translation start site. Thus, the indel may be functionally neutral.
(0.17 MB TIF)

Figure S4 Conservation of coding indels with size 3n that are not located at the N- or C-termini of the protein, near exon boundaries, or in tandem repeats. The x-axis is the percentile of amino acid conservation at the indel's location relative to all of the positions in the protein. A low percentile indicates that the indel is located at a nonconserved position in the protein. A high percentile indicates that the indel is located at a conserved position, relative to all other positions in the protein.
(0.21 MB TIF)

Figure S5 A) The proportion of exons in copy number regions, tandem repeats, and RepeatMasker regions. The solid bars represent the percentages observed for exons missing from the HuRef assembly; the hatched bars represents the percentages observed for all exons. B) The coverage of exons missing from the HuRef assembly (solid line) has a bimodal distribution. As a control, the coverage of exons randomly selected from all exons is shown (dashed line) and is normally distributed.
(0.28 MB TIF)

Figure S6 A) Comparison of the allele frequencies for nsSNPs in disease genes versus nsSNPs in all genes. The distributions are similar and not significantly different (p = 0.97). B) The percentage of nsSNPs predicted to affect protein function in disease genes is similar to nsSNPs in all genes.
(0.31 MB TIF)

Figure S7 Genes involved in the melanoma pathway [99], overlaid with HuRef variants. For each gene symbol, a pie chart represents the HuRef variants found in or within 1 kb of the gene. Variants that were in coding, UTR, and conserved regions were counted. If a gene has no pie chart, no variants were found in these regions. The size of the pie chart corresponds to the number of variants, and colors correspond to the fraction of variants for each type. Of the 67 variants found in/near these genes, only 1 was nonsynonymous. The nsSNP was in TP53, and it is frequent in the CEU population (MAF = 0.23). 63% of the variants were in conserved regions.
(0.49 MB TIF)

Table S1 Pre-termination codon SNPs.
(0.06 MB XLS)

Table S2 Variants validated by targeted resequencing.
(0.03 MB XLS)

Table S3 nsSNPs in disease genes.
(0.40 MB XLS)

Table S4 Indels in disease genes.
(0.03 MB XLS)

Table S5 Diversity rates.
(0.04 MB XLS)

Text S1 Supplemental text.
(0.03 MB DOC)
