# Supplementary figures and images for: Genetic Variation in an Individual Human Exome
Source: PLoS Genet. 2008 Aug 15;4(8):e1000160. doi: 10.1371/journal.pgen.1000160 (PMC2493042; doi:10.1371/journal.pgen.1000160)

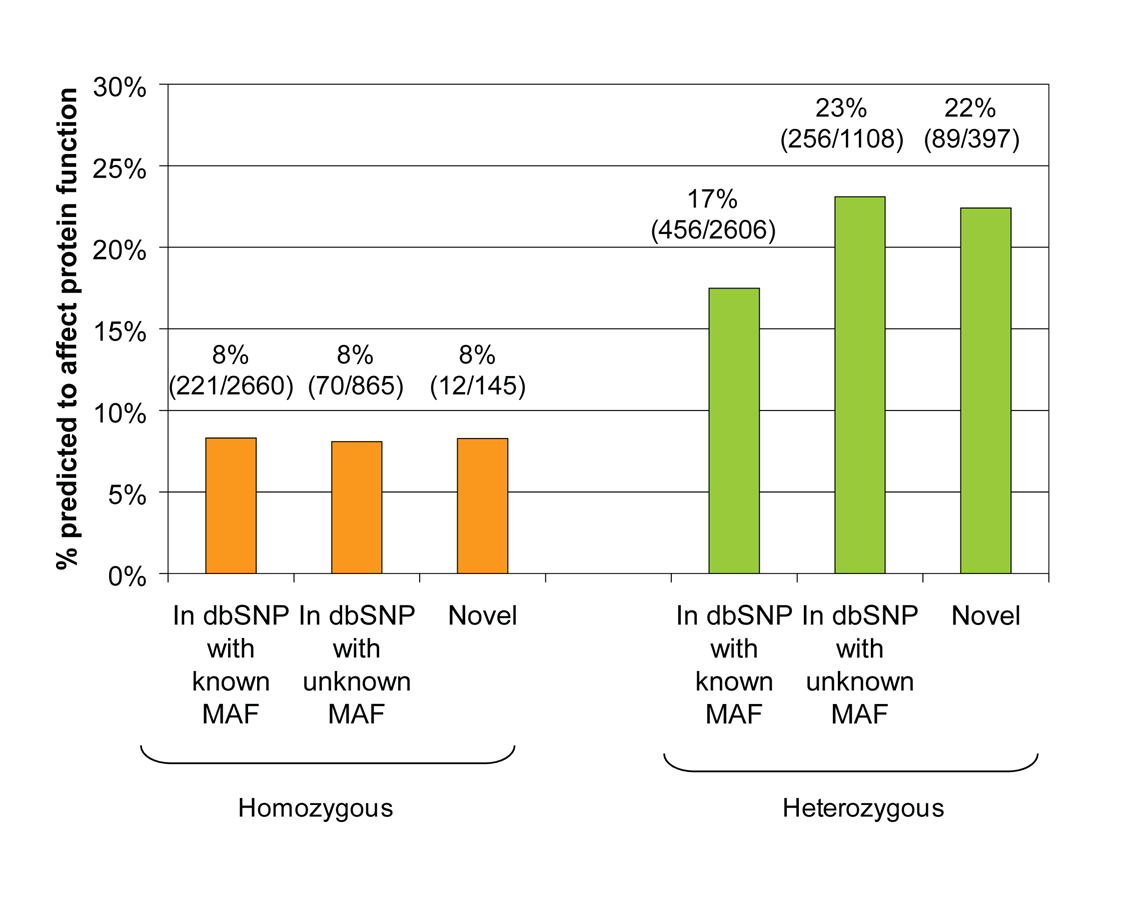

Supplement: Figure S1 — Protein-affecting predictions for nsSNPs that are novel or in dbSNP with unknown allele frequencies. When categorized by zygosity, the percentage of predicted-protein-affecting nsSNPs is similar between the different categories for homozygous nsSNPs, but not for the heterozygous nsSNPs. (0.25 MB TIF) [file pgen.1000160.s001.tif]

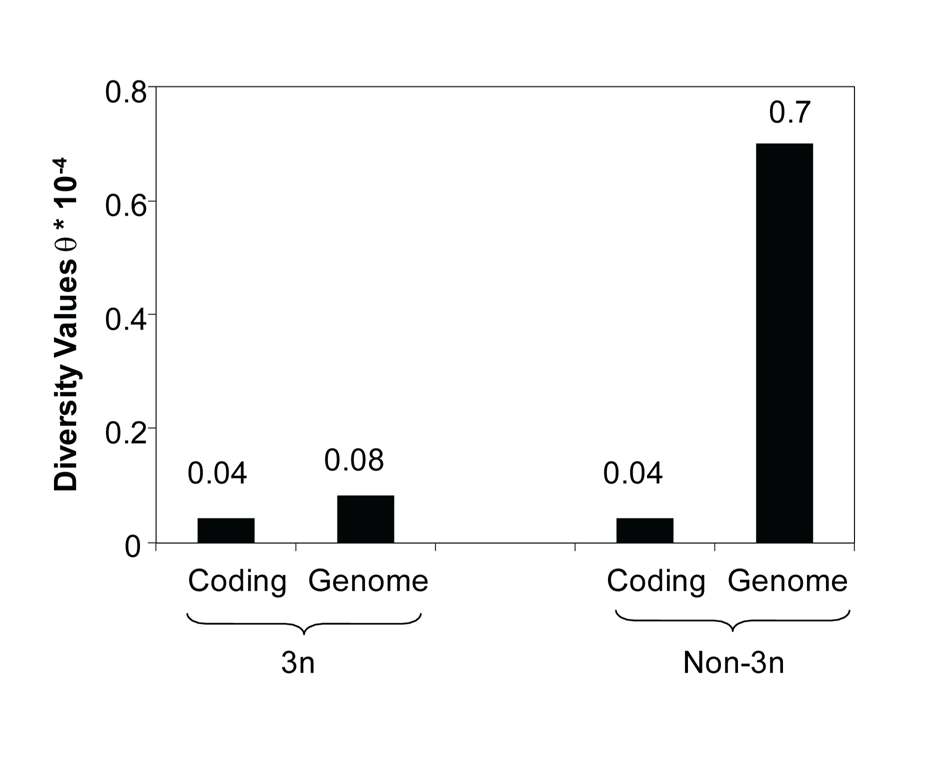

Supplement: Figure S2 — Diversity rates for indels, based on size. (0.12 MB TIF) [file pgen.1000160.s002.tif]

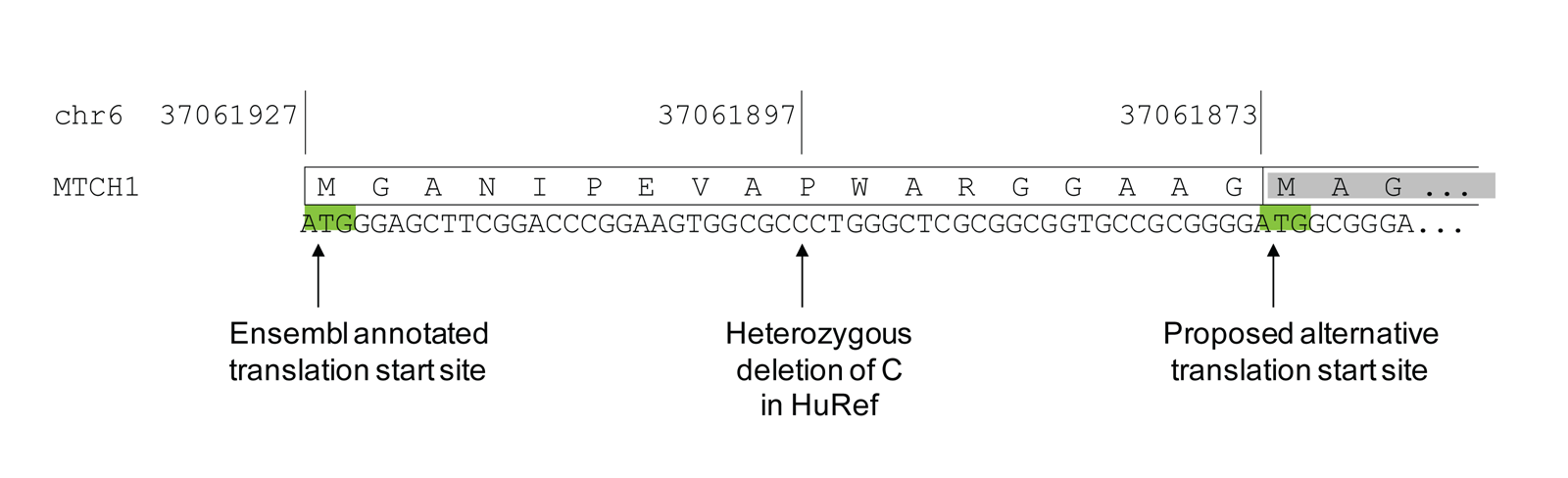

Supplement: Figure S3 — An example of an indel that occurs at the N-terminus of the gene MTCH1. This indel occurs in the first exon, about 30 bp after the translation start site. A start codon just downstream of the indel may serve as an alternative translation start site. Thus, the indel may be functionally neutral. (0.17 MB TIF) [file pgen.1000160.s003.tif]

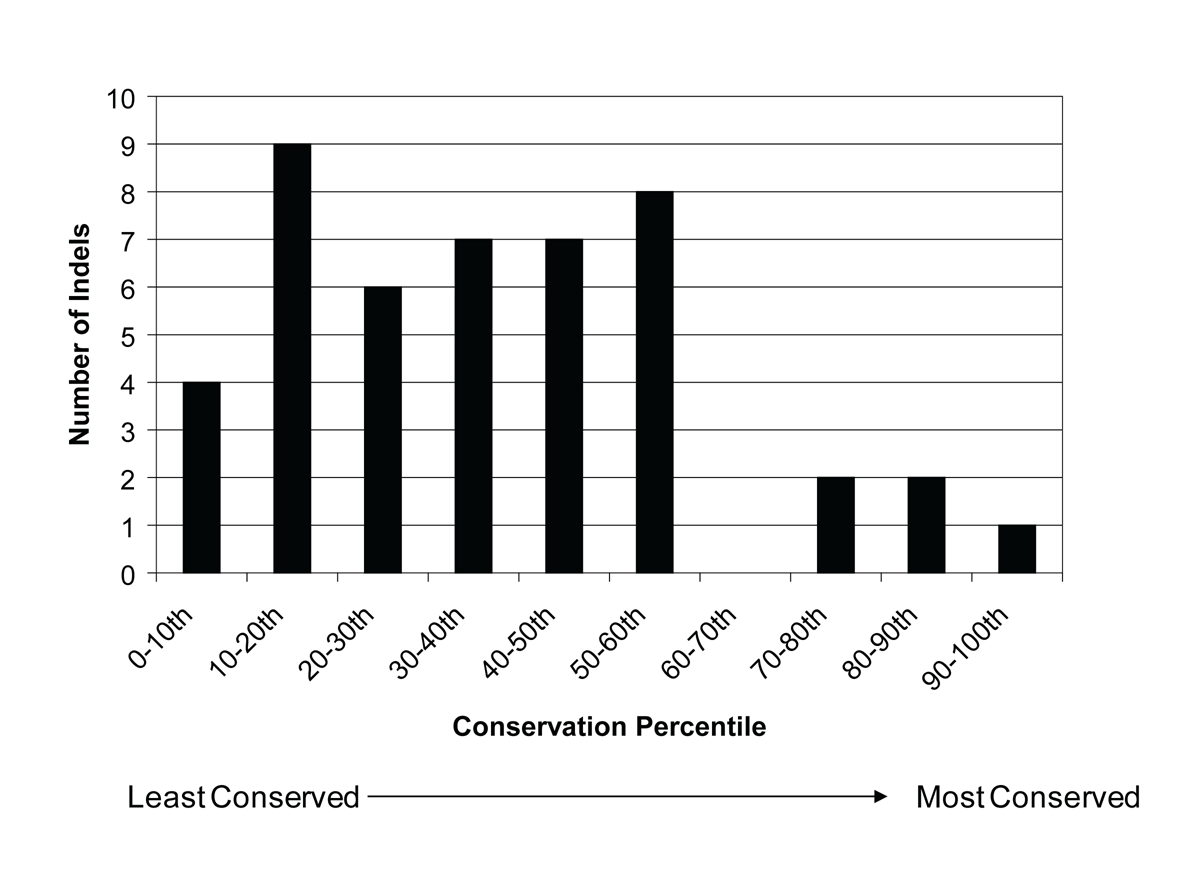

Supplement: Figure S4 — Conservation of coding indels with size 3n that are not located at the N- or C-termini of the protein, near exon boundaries, or in tandem repeats. The x-axis is the percentile of amino acid conservation at the indel's location relative to all of the positions in the protein. A low percentile indicates that the indel is located at a nonconserved position in the protein. A high percentile indicates that the indel is located at a conserved position, relative to all other positions in the protein. (0.21 MB TIF) [file pgen.1000160.s004.tif]

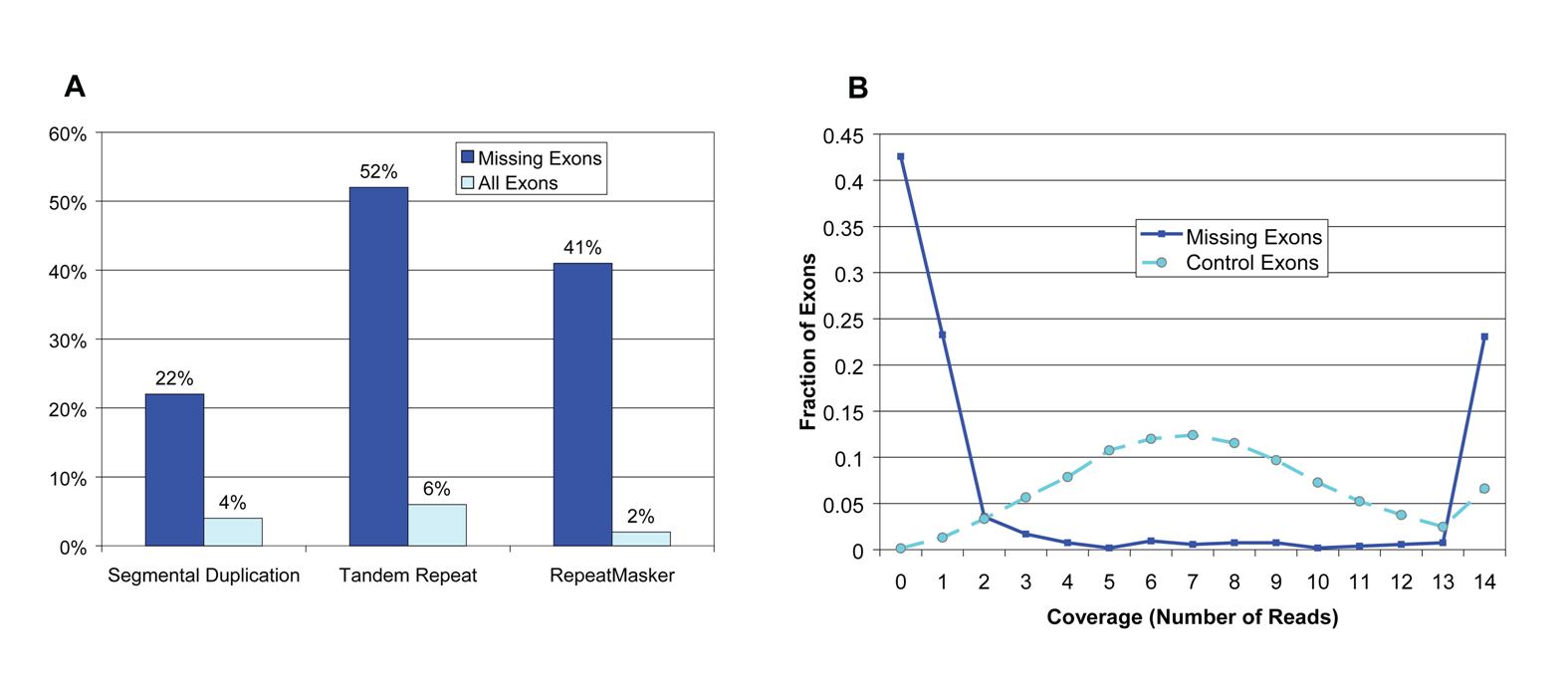

Supplement: Figure S5 — A) The proportion of exons in copy number regions, tandem repeats, and RepeatMasker regions. The solid bars represent the percentages observed for exons missing from the HuRef assembly; the hatched bars represents the percentages observed for all exons. B) The coverage of exons missing from the HuRef assembly (solid line) has a bimodal distribution. As a control, the coverage of exons randomly selected from all exons is shown (dashed line) and is normally distributed. (0.28 MB TIF) [file pgen.1000160.s005.tif]

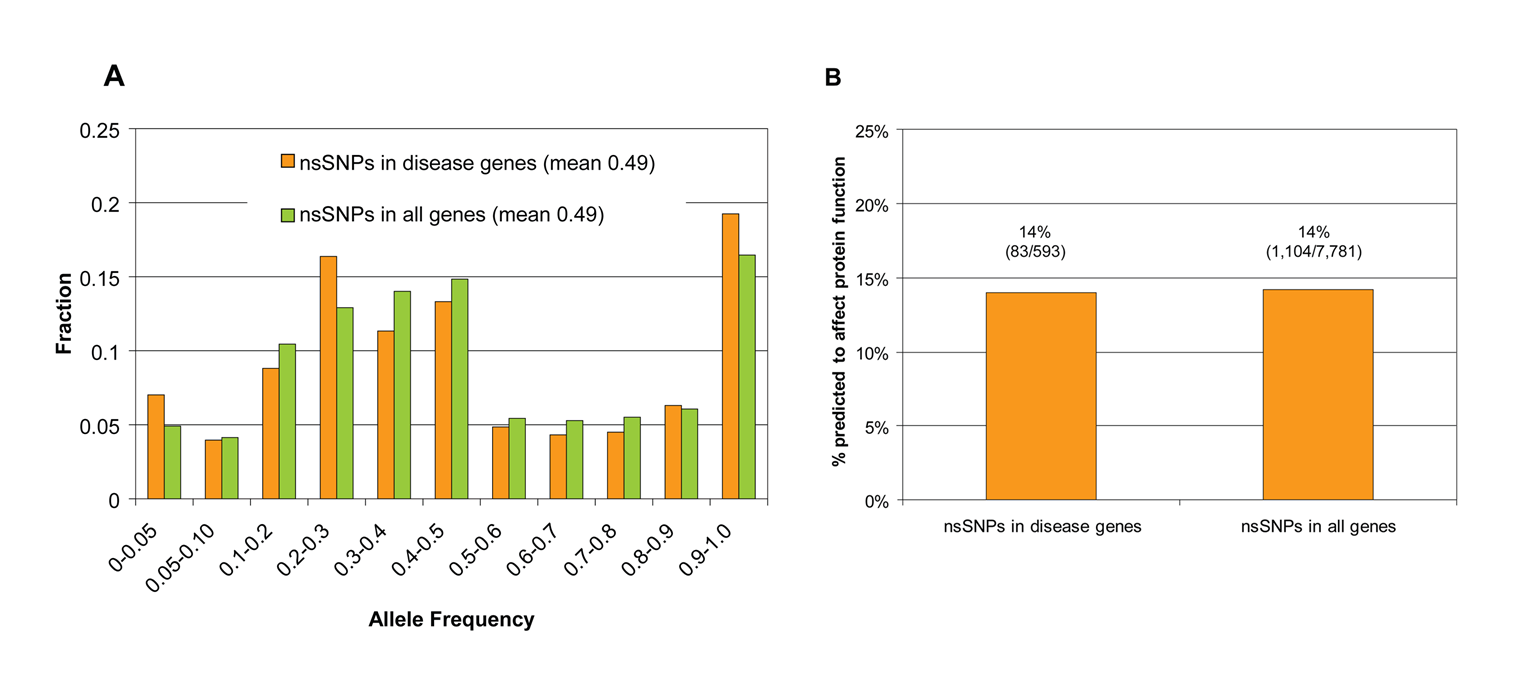

Supplement: Figure S6 — A) Comparison of the allele frequencies for nsSNPs in disease genes versus nsSNPs in all genes. The distributions are similar and not significantly different (p = 0.97). B) The percentage of nsSNPs predicted to affect protein function in disease genes is similar to nsSNPs in all genes. (0.31 MB TIF) [file pgen.1000160.s006.tif]

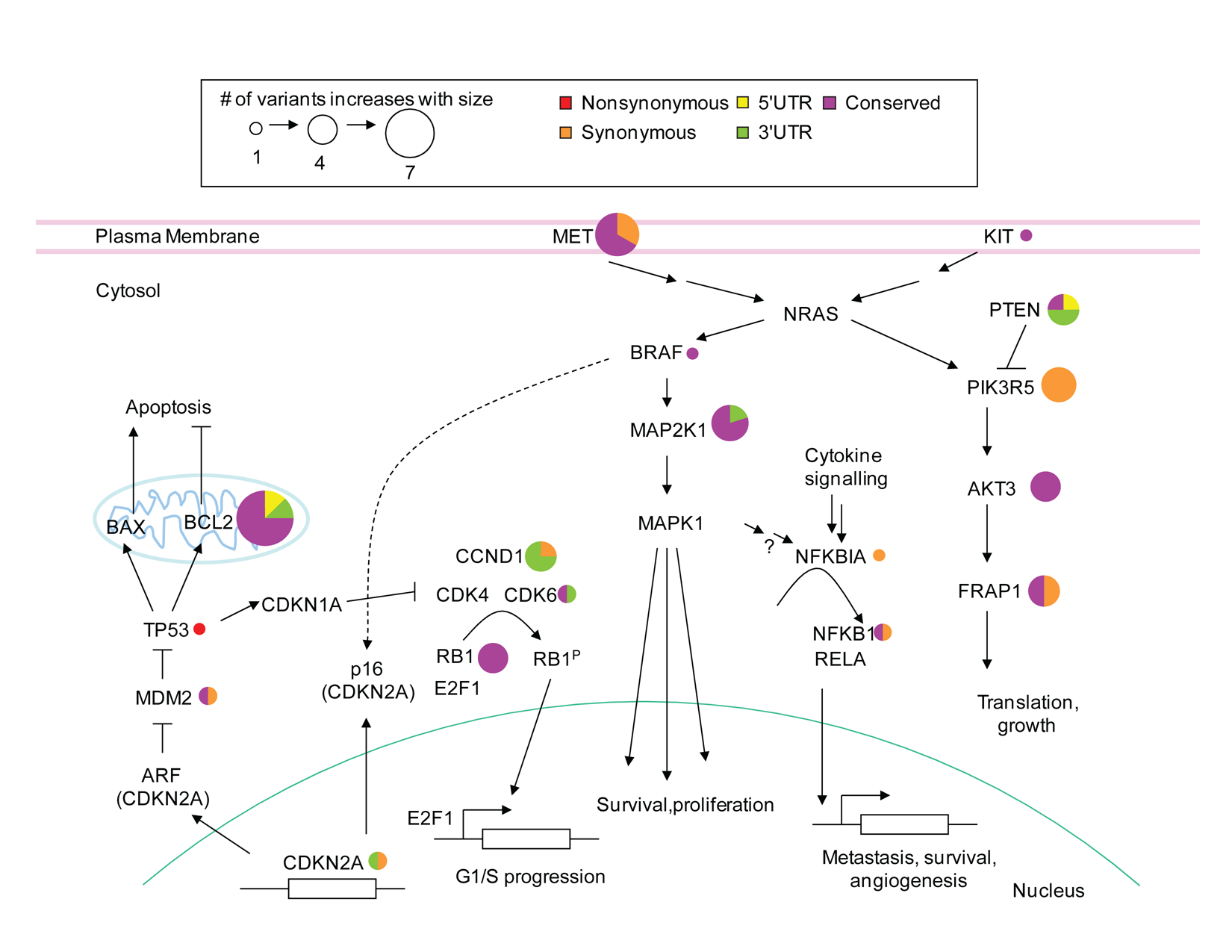

Supplement: Figure S7 — Genes involved in the melanoma pathway [99], overlaid with HuRef variants. For each gene symbol, a pie chart represents the HuRef variants found in or within 1 kb of the gene. Variants that were in coding, UTR, and conserved regions were counted. If a gene has no pie chart, no variants were found in these regions. The size of the pie chart corresponds to the number of variants, and colors correspond to the fraction of variants for each type. Of the 67 variants found in/near these genes, only 1 was nonsynonymous. The nsSNP was in TP53, and it is frequent in the CEU population (MAF = 0.23). 63% of the variants were in conserved regions. (0.49 MB TIF) [file pgen.1000160.s007.tif]
