# Supplementary material for: Genetic Variation in an Individual Human Exome
Source: PLoS Genet. 2008 Aug 15;4(8):e1000160. doi: 10.1371/journal.pgen.1000160 (PMC2493042; doi:10.1371/journal.pgen.1000160)
Supplement: Text S1 — Supplemental text. (0.03 MB DOC) [file pgen.1000160.s013.doc]

**Text S1**

**Characterizing SNPs with Unknown Allele Frequencies**

We wanted to determine the effect of SNPs with unknown allele frequencies (AFs). These are defined by two categories: 1) the novel nsSNPs which are absent from dbSNP and 2) nsSNPs found in dbSNP but with unknown MAF. 19% of the novel nsSNPs and 17% of the dbSNP SNPs with unknown AF were predicted to affect function. These percentages are higher than what is observed for SNPs with known AFs, and the difference is due to the heterozygous SNPs and not the homozygous nsSNPs (Figure S1). Because a higher proportion of the heterozygous nsSNPs with unknown AF are predicted to be affect function, we expect that a higher proportion are undergoing negative selection compared to the nsSNPs with known AFs, and hence, a larger proportion of these will be rare. We were able to confirm this expectation by determining the minor allele frequencies of 26 heterozygous novel nsSNPs (see Methods and Table S2). The mean MAF of the novel SNPs was 0.09 and three-fourths of the SNPs were rare. In contrast, the mean MAF for heterozygous nsSNPs that are in dbSNP with known MAFs is 0.26, with 9% of the nsSNPs being rare.

**Genes with missing or partial exons**

We identified 1,454 exons in 1,046 genes where at least half of the coding exon’s sequence was missing from the HuRef assembly. Almost a third of the genes mapped to chrX or chrY. The observation that many of the missing exons were located on sex chromosomes is likely due to low sequence coverage; in the HuRef assembly, sex chromosomes have half the read depth than autosomal chromosomes [1]. After removing the genes that reside on sex chromosomes, there were 719 genes with missing or partial exons remaining.  The missing exons in these genes overlapped with copy number regions, tandem repeats, and regions identified by RepeatMasker at a higher proportion than expected by chance (Figure S5a). 96% of the missing exons were in at least one of these three region types. This illustrates that genes that appear to be partially missing are more likely due to difficulties in genome assembly rather than the human individual truly missing part of a gene. We further verified this by re-mapping the reads from the HuRef assembly to the missing exons (Figure S5b). The missing exons show a bimodal distribution with either very few reads or many reads. This reflects that genes with “missing” exons are most likely due to low coverage or assembly issues with repetitive regions. This was confirmed by resequencing a subset of the missing exons in the HuRef sample and validating that in fact, most of the “missing exons” are present in the HuRef sample (see Methods).

**Coding SNPs in OMIM**

In Table 2, we list the HuRef nsSNPs with dbSNP identifiers that are also found in the disease database OMIM. These SNPs were obtained using a batch query with “OMIMSNP” as the handle. The OMIM database contains many amino acid substitutions that have been found in patients with disease. We required the dbSNP status because OMIM variant entries only provide amino acid locations with respect to the protein. The protein locations are relative to the protein sequence, but the exact protein sequence which is used as a reference is unknown. For genes with multiple transcripts (and hence multiple protein isoforms) or genes that have undergone protein sequence revision since the amino acid substitution was deposited in OMIM, it is difficult to obtain the correct position. Taken together, the use of OMIM’s protein position alone is not sufficiently precise without extensive manual analysis. The dbSNP status provides chromosomal coordinates so we can ensure that the HuRef variant corresponds to the correct OMIM entry.

**Coding Indels In Disease Genes**

There are three non-3n indels in disease genes. Two of these indels appear to be annotation issues. One indel is in the first exon of the gene ZFYVE19, and there is a downstream start codon that could serve as an alternative translation start site. The second is likely to be a gene annotation problem for NDUFS7, where the indel lies in a region which Ensembl predicts as coding, but this disagrees with RefSeq annotation which designates this region as intronic. Thus we surmise that these two cases are likely to be functionally neutral and suggest that close inspection of coding indels can improve gene annotation. The third non-3n indel is in ACOX2 and is described in the main text.
